# Curiosities of Foods

**Published:** 1888-06

**Authors:** 


### CURIOSITIES OF FOODS.

The old saying that what is one man's meat is another man's poison is realized in the opposite tastes of people. The Turks shudder at the thought of eating oysters. The digger Indians of the Pacific Coast rejoiced in the great locust swarms of 1875 as a dispensation of the Great Spirit, and laid in a store of dried locust powder sufficient to last them for several years. The French will eat frogs, snails and the diseased livers of geese, but draw the line at aligators. Buckland declares the taste of boaconstrictors to be good and much like veal. Quass, the fermented cabbage-water of the Russians, is their popular tipple. It is described as resembling a mixture of stale fish and soapsuds in taste, yet, next to beer, it has more votaries than any other fermented beverage. A tallow candle washed down with quass forms a meal that it would be hard to be thankful for.

In Canton and other Chinese cities rats are sold at the rate of 50 cents a dozen, and the hindquarters of the dog are hung up in the butchers' shop alongside of mutton and lamb, but command a higher price. The edible birds' nests of the Chinese are worth twice their weight in silver, the finest variety selling for as much as $30 a pound. The negroes of the West Indies eat baked snakes and palm worms fried in fat, but they cannot be induced to eat stewed rabbits. In Mexico parrots are eaten, but they are rather tough. The Guachos of the Argentine Republic are in the habit of hunting skunks for the sake of their flesh. The octopus, or devil fish, when boiled and then roasted, is eaten in Corsica and esteemed a delicacy. In the Pacific Islands and West Indies lizard eggs are eaten with gusto.

The natives of the Antilles eat alligator eggs, and the eggs of the turtle are popular everywhere, though up to the commencement of the last century turtle was only eaten by the poor of Jamaica. Ants are eaten by various nations. In Brazil they are served with a resinous sauce, and in Africa they are stewed in grease or butter. The East Indians catch them in pits and carefully wash them in handfuls like raisins. In Siam a curry of ant eggs is a costly luxury. The Cingales eat the bees after robbing them of their honey. Caterpillars and spiders are dainties to the African bushmen. After they have wound the silk from the cocoon, the Chinese eat the chrysalis of the silkworm. Spiders roasted are a sort of dessert with the New Caledonians.—*Pacific Record.*

---

Bmtter.—Duolaux has published interesting results of his investigations. According to them, butter consists of 93 per cent. oleine, margarine and stearine. 4.4 per cent. butyrine, 2.5 caproine, 0.1 per cent. caprylinе and caprynine, and, as stated by Chevieul, the centenarian, contains even when fresh some free butyric acid. In beginning rancescence, butyrine is first to be decomposed, as a result of the spontaneous decomposability of glycerids ; it is retarded by salts, accelerated by water, light and air. Butter absorbs oxygen, at first without a corresponding formation of carbonic acid, which is formed later together with formic acid.—
*La Pharmacien*

